# Correction: Women’s visibility in academic seminars: Women ask fewer questions than men

**DOI:** 10.1371/journal.pone.0212146

**Published:** 2019-02-06

**Authors:** Alecia J. Carter, Alyssa Croft, Dieter Lukas, Gillian M. Sandstrom

There is an error in the author byline. The third author, Dr. Dieter Lukas, should be included as a Corresponding Author. Dr. Lukas’ email address is: dieter_lukas@eva.mpg.de.
