# Supplementary material for: Women’s visibility in academic seminars: Women ask fewer questions than men
Source: PLoS One. 2018 Sep 27;13(9):e0202743. doi: 10.1371/journal.pone.0202743 (PMC6159863; doi:10.1371/journal.pone.0202743)
Supplement: S1 File — The list of questions and the flow of the Qualtrics survey. (PDF) [file pone.0202743.s001.pdf]

S1 File for: Women's visibility in academic seminars: women ask fewer questions than men

Survey questions and survey flow

Q1 Thank you for taking the time to participate! In this study, you will be asked some questions about your attendance and participation in academic seminars and the culture around participation in seminars in your department, before we ask some questions about your demography. The study will take fewer than 10 minutes to complete. You can withdraw your participation at any time during the study and are never obliged to answer a question. Your privacy is very important. We will ask you for some demographic information, but nothing that could identify you. We are committed to open science, so the data that we collect will be made available online, for use by other researchers (e.g., at https://osf.io/). Results from this study will be presented in academic publications. Results are normally presented in terms of groups of individuals. If any individual data were presented, the data would be anonymous, without any means of identifying the individuals involved. This project has received ethical approval from the Departmental Psychology Ethics Committee of the University of Essex. If you have any questions you'd like to ask before starting the survey, please feel free to contact Dr. Gillian Sandstrom at gsands@essex.ac.uk, Dr Alecia Carter at ac854@cam.ac.uk, Dr Dieter Lukas at dl384@cam.ac.uk or Dr Alyssa Croft at alyssac@email.arizona.edu. Consent to Participate: I have read and understood the consent form. I have had sufficient time to consider the information provided and to ask for advice if necessary. I have had the opportunity to ask questions and have had satisfactory responses to my questions. I understand that all of the information collected will be kept confidential and that the results will be made publically available. I understand that my participation in this study is voluntary and that I am completely free to refuse to participate or to withdraw from this study at any time. I understand that I am not waiving any of my legal rights as a result of agreeing to this consent form. If you consent, please click the 'I consent' button below and then click the arrow to begin the survey.

- ❍ I consent (1)

If I consent Is Not Selected, Then Skip To End of Survey

Q2 For this survey, we define a seminar as a public presentation at an academic institution attended by students and faculty. On average, how many of these types of seminars have you attended?

- ❍ >1 per week (1)
- ❍ Weekly (2)
- ❍ Fortnightly/Bi-weekly (3)
- ❍ Monthly (4)
- ❍ A few times per year (5)

Q3 Do you ask questions in:

- ❍ All seminars? (1)
- ❍ Most seminars? (2)
- ❍ Some seminars? (3)
- ❍ Few seminars? (4)
- ❍ No seminars? (5)

Q4 Have you ever NOT asked a question when you wanted to?

❍ Yes (sometimes I don't ask questions, even when I have one) (1)

❍ No (I always ask the questions that I want to ask) (2)

If No (I always ask the questi... Is Selected, Then Skip To If you ask questions at seminars, wha...

Q5 How important is each of these factors in stopping you from asking a question?

	Not at all important (1)	Slightly important (2)	Moderately important (3)	Very important (4)	Extremely important (5)
Not enough time (1)	❍	❍	❍	❍	❍
Worried that I had misunderstood the content (2)	❍	❍	❍	❍	❍
Couldn't work up the nerve (3)	❍	❍	❍	❍	❍
Not sure whether the question was appropriate (4)	❍	❍	❍	❍	❍
Not my field (5)	❍	❍	❍	❍	❍
The speaker was too eminent/intimidating (6)	❍	❍	❍	❍	❍
Worried that I was not clever enough to ask a good question (7)	❍	❍	❍	❍	❍
I was meeting the speaker later / asked after the talk had ended (8)	❍	❍	❍	❍	❍
Other (please specify): (9)	❍	❍	❍	❍	❍

Q7 To what extent would each of these factors encourage you to ask more questions?

	Wouldn't help at all (1)	Wouldn't help much (2)	Might help a bit (3)	Would help a lot (4)	Would make a huge difference (5)
A longer time to formulate the question (1)	❍	❍	❍	❍	❍
A chance to ask in person (2)	❍	❍	❍	❍	❍
Nicer speakers (3)	❍	❍	❍	❍	❍
More welcoming host (4)	❍	❍	❍	❍	❍
Confidence (5)	❍	❍	❍	❍	❍
Seniority (6)	❍	❍	❍	❍	❍
Having a moderator to ask the questions (7)	❍	❍	❍	❍	❍
Moderator doing a better job engaging whole audience (8)	❍	❍	❍	❍	❍
Other (please specify): (9)	❍	❍	❍	❍	❍

Q6 If / when you ask questions at seminars, what has been your main motivation? (Check all that apply.)

- ❑ Interested in subject (1)
- ❑ Feel you spotted a mistake (2)
- ❑ Need for clarification (3)
- ❑ I feel it's part of my role (e.g., to act as a model for more junior academics) (4)
- ❑ To establish a connection with a particular speaker (5)

Q28 What factors do you think play a role in who asks questions?Does Seniority play a role in who asks questions in seminars?

- ❍ Senior audience members ask more questions (1)
- ❍ Junior audience members ask more questions (2)
- ❍ Senior and Junior audience members ask about the same amount of questions (3)

Q30 Does Confidence play a role in who asks questions in seminars?

- ❍ Confident people ask more questions (1)
- ❍ Not confident people ask more questions (2)
- ❍ Confident and Not confident people ask about the same amount of questions (3)

Q27 Does Extraversion play a role in who asks questions in seminars?

- ❍ Introverted people ask more questions (1)
- ❍ Extraverted people ask more questions (2)
- ❍ Introverted and Extraverted people ask about the same amount of questions (3)

Q29 Does Gender play a role in who asks questions in seminars?

- ❍ Women ask more questions (1)
- ❍ Men ask more questions (2)
- ❍ Men and Women ask about the same amount of questions (3)

Q31 Does Competence play a role in who asks questions in seminars?

- ❍ Competent people ask more questions (1)
- ❍ Incompetent people ask more questions (2)
- ❍ Competent and Incompetent people ask about the same amount of questions (3)

Q28 Do other factors play a role in who asks questions during seminars?

- ❍ No (4)
- ❍ Maybe (5) ____________________
- ❍ Yes (6) ____________________

Answer If Does Gender play a role in who asks questions in seminars? Men ask more questions Is Selected Or Does Gender play a role in who asks questions in seminars? Women ask more questions Is Selected

Q34 You indicated that gender plays a role in who asks questions. How important do you think each of these factors is in preventing the gender asking fewer questions from asking more questions?

	Not at all important (1)	Slightly important (2)	Moderately important (3)	Very important (4)	Extremely important (5)
Worry that they misunderstand the content (1)	❍	❍	❍	❍	❍
Can't work up the nerve (2)	❍	❍	❍	❍	❍
Are unsure that their questions are appropriate (3)	❍	❍	❍	❍	❍
Feel they are not an expert (4)	❍	❍	❍	❍	❍
Feel intimidated by the speaker (5)	❍	❍	❍	❍	❍
Believe that they are not clever enough to ask a good question (6)	❍	❍	❍	❍	❍
Ask questions after the seminar is over (7)	❍	❍	❍	❍	❍
Other (please specify): (8)	❍	❍	❍	❍	❍

Q14 How much time is usually provided for questions after seminars?

❍ (1)
❍ 5-10 min (2)
❍ 11-15 min (3)
❍ 16-30 min (4)
❍ >30 min (5)

Q15 How many people usually attend your departmental seminars?

- (1)
- 15-30 (2)
- 31-45 (3)
- >45 (4)

Q27 How easy is it to meet invited speakers informally?

- Extremely easy (1)
- Easy enough (2)
- Not easy or difficult (3)
- A bit difficult (4)
- It's not possible (5)

Q17 What is the culture around meeting speakers in your department?

- Speakers meet only with the host (1)
- Speakers meet with relevant faculty (2)
- Anyone can sign up to meet a speaker (3)
- Everyone is actively encouraged to meet speakers, and speakers have organized events (e.g. lunch with PhD students) (4)

Q13 In your department, what percentage of the permanent faculty are women?

- (1)
- 10-25% (2)
- 26-50% (3)
- 51-75% (4)
- 76-100% (5)
- I don't know (6)

Q28 In your department, what percentage of the graduate/PhD students are women?

- (1)
- 10-25% (2)
- 26-50% (3)
- 51-75% (4)
- 76-100% (5)
- I don't know (6)

Q11 What is your subject? (Please choose one from the list.)

- Accounting and Finance (1)
- Anthropology (2)
- Archaeology (3)
- Art History (4)
- Biochemistry (5)
- Biological Sciences (6)
- Biomedical Sciences (7)
- Business and Management (8)
- Chemistry (9)
- Classics and Ancient History (10)
- Computer Science and IT (11)
- Criminology (12)
- Drama (13)
- Earth Sciences (14)
- Economics (15)
- Education (16)
- Engineering (17)
- English language (18)
- English literature (19)
- Environmental Science (20)
- Film Studies (21)
- Geography (22)
- Geology (23)
- History (24)
- Human Sciences (25)
- International studies (26)
- Law (27)
- Liberal Arts (28)
- Linguistics (29)
- Materials Science (30)
- Mathematics (31)
- Medicine (32)
- Modern Languages (33)
- Music (34)
- Natural Sciences (35)
- Neuroscience (36)
- Philosophy (37)
- Physics and Astronomy (38)
- Politics and International Relations (39)
- Psychology (40)
- Social Sciences (41)
- Sociology and Criminology (42)
- Sport and Health Sciences (43)

- ❍ Theology and Religion (44)
- ❍ Something else (45)

Q18 What career stage are you?

- ❍ Undergraduate student (1)
- ❍ Postgraduate student (e.g., Masters, PhD) (2)
- ❍ Postdoctoral researcher (3)
- ❍ Research fellow (4)
- ❍ Faculty (5)
- ❍ Other (please specify) (6) ____________________

Q19 How long have you been at this stage?

- ❍ (1)
- ❍ 1-2 years (2)
- ❍ 3-5 years (3)
- ❍ 6-10 years (4)
- ❍ >10 years (5)

Q21 In which country is your current institution?

- Afghanistan (1)
- Albania (2)
- Algeria (3)
- Andorra (4)
- Angola (5)
- Antigua and Barbuda (6)
- Argentina (7)
- Armenia (8)
- Australia (9)
- Austria (10)
- Azerbaijan (11)
- Bahamas (12)
- Bahrain (13)
- Bangladesh (14)
- Barbados (15)
- Belarus (16)
- Belgium (17)
- Belize (18)
- Benin (19)
- Bhutan (20)
- Bolivia (21)
- Bosnia and Herzegovina (22)
- Botswana (23)
- Brazil (24)
- Brunei Darussalam (25)
- Bulgaria (26)
- Burkina Faso (27)
- Burundi (28)
- Cambodia (29)
- Cameroon (30)
- Canada (31)
- Cape Verde (32)
- Central African Republic (33)
- Chad (34)
- Chile (35)
- China (36)
- Colombia (37)
- Comoros (38)
- Congo, Republic of the... (39)
- Costa Rica (40)
- Côte d'Ivoire (41)
- Croatia (42)
- Cuba (43)

- Cyprus (44)
- Czech Republic (45)
- Democratic People's Republic of Korea (46)
- Democratic Republic of the Congo (47)
- Denmark (48)
- Djibouti (49)
- Dominica (50)
- Dominican Republic (51)
- Ecuador (52)
- Egypt (53)
- El Salvador (54)
- Equatorial Guinea (55)
- Eritrea (56)
- Estonia (57)
- Ethiopia (58)
- Fiji (59)
- Finland (60)
- France (61)
- Gabon (62)
- Gambia (63)
- Georgia (64)
- Germany (65)
- Ghana (66)
- Greece (67)
- Grenada (68)
- Guatemala (69)
- Guinea (70)
- Guinea-Bissau (71)
- Guyana (72)
- Haiti (73)
- Honduras (74)
- Hong Kong (S.A.R.) (75)
- Hungary (76)
- Iceland (77)
- India (78)
- Indonesia (79)
- Iran, Islamic Republic of... (80)
- Iraq (81)
- Ireland (82)
- Israel (83)
- Italy (84)
- Jamaica (85)
- Japan (86)
- Jordan (87)

- Kazakhstan (88)
- Kenya (89)
- Kiribati (90)
- Kuwait (91)
- Kyrgyzstan (92)
- Lao People's Democratic Republic (93)
- Latvia (94)
- Lebanon (95)
- Lesotho (96)
- Liberia (97)
- Libyan Arab Jamahiriya (98)
- Liechtenstein (99)
- Lithuania (100)
- Luxembourg (101)
- Madagascar (102)
- Malawi (103)
- Malaysia (104)
- Maldives (105)
- Mali (106)
- Malta (107)
- Marshall Islands (108)
- Mauritania (109)
- Mauritius (110)
- Mexico (111)
- Micronesia, Federated States of... (112)
- Monaco (113)
- Mongolia (114)
- Montenegro (115)
- Morocco (116)
- Mozambique (117)
- Myanmar (118)
- Namibia (119)
- Nauru (120)
- Nepal (121)
- Netherlands (122)
- New Zealand (123)
- Nicaragua (124)
- Niger (125)
- Nigeria (126)
- Norway (127)
- Oman (128)
- Pakistan (129)
- Palau (130)
- Panama (131)

- Papua New Guinea (132)
- Paraguay (133)
- Peru (134)
- Philippines (135)
- Poland (136)
- Portugal (137)
- Qatar (138)
- Republic of Korea (139)
- Republic of Moldova (140)
- Romania (141)
- Russian Federation (142)
- Rwanda (143)
- Saint Kitts and Nevis (144)
- Saint Lucia (145)
- Saint Vincent and the Grenadines (146)
- Samoa (147)
- San Marino (148)
- Sao Tome and Principe (149)
- Saudi Arabia (150)
- Senegal (151)
- Serbia (152)
- Seychelles (153)
- Sierra Leone (154)
- Singapore (155)
- Slovakia (156)
- Slovenia (157)
- Solomon Islands (158)
- Somalia (159)
- South Africa (160)
- Spain (161)
- Sri Lanka (162)
- Sudan (163)
- Suriname (164)
- Swaziland (165)
- Sweden (166)
- Switzerland (167)
- Syrian Arab Republic (168)
- Tajikistan (169)
- Thailand (170)
- The former Yugoslav Republic of Macedonia (171)
- Timor-Leste (172)
- Togo (173)
- Tonga (174)
- Trinidad and Tobago (175)

- ❍ Tunisia (176)
- ❍ Turkey (177)
- ❍ Turkmenistan (178)
- ❍ Tuvalu (179)
- ❍ Uganda (180)
- ❍ Ukraine (181)
- ❍ United Arab Emirates (182)
- ❍ United Kingdom of Great Britain and Northern Ireland (183)
- ❍ United Republic of Tanzania (184)
- ❍ United States of America (185)
- ❍ Uruguay (186)
- ❍ Uzbekistan (187)
- ❍ Vanuatu (188)
- ❍ Venezuela, Bolivarian Republic of... (189)
- ❍ Viet Nam (190)
- ❍ Yemen (191)
- ❍ Zambia (192)
- ❍ Zimbabwe (193)

Q12 What is your gender?
- ❍ Male (1)
- ❍ Female (2)
- ❍ Transgender (3)
- ❍ Prefer not to say (4)

Q22 The study's aims: This study was designed to help us understand why there is a bias in the gender ratio of academics that attend and ask questions during seminars. Our preliminary research shows that more women attend seminars than men, but they ask fewer questions. From your answers, we would like to make recommendations that will lead to an improved visibility of women in academia through fostering an environment that promotes women's participation in regular academic events. The last thing that we want to ask you is not to share your knowledge about the true purpose of this study. We will be running this study for several weeks. As you can imagine, it would be very difficult for us to collect accurate information if people knew about the true purpose of this study beforehand. Consequently, we would appreciate if you do not discuss the true aim of this survey with others. Thank you so much for participating in this research. Without your help we would be unable to test our hypotheses and gather the necessary data. In case you are interested in the findings of the survey, we will be updating this website once the survey is completed http://academicseminarparticipation.strikingly.com/ If you have any questions, please contact any of the investigators on the project: Dr. Gillian Sandstrom (gsands@essex.ac.uk); Dr Alecia Carter (ac854@cam.ac.uk); Dr Dieter Lukas (dl384@cam.ac.uk); Dr Alyssa Croft (alyssac@email.arizona.edu).
