# Supplementary material for: Women’s visibility in academic seminars: Women ask fewer questions than men
Source: PLoS One. 2018 Sep 27;13(9):e0202743. doi: 10.1371/journal.pone.0202743 (PMC6159863; doi:10.1371/journal.pone.0202743)
Supplement: S2 File — The instructions that were distributed to the observers who agreed to collect data at their local seminars. (PDF) [file pone.0202743.s002.pdf]

S2 File for: Women's visibility in academic seminars: women ask fewer questions than men
Instructions for collecting observational data

Brief background: The attrition of women in academia is a major concern, particularly in Science, Technology, Engineering, and Mathematics subjects. One factor that may contribute to the attrition of women is the lack of visibility of women in academia, which may result in women feeling like they do not belong. One place where academics regularly meet and see other academics is at departmental seminars. Our preliminary research suggests that more women attend departmental seminars than men, but ask disproportionally fewer questions than male attendees. However, thus far, we have collected data only at seminars in the University of Cambridge, which may not be representative of trends elsewhere. Our aim is to broaden our sample of seminars to other subjects, institutions, and countries to determine whether the trend is similar. We would be very grateful if you would help by collecting data in your department. We hope that this will not be onerous, as it will involve only a bit of counting during seminars you're already attending during one semester (and staying until the very end of the seminar!). As it would be better for our analyses if we could control for departmental differences using random effects, we would like a sample of about 3-4 seminars per department i.e. 3 or 4 data from you. This is not absolutely necessary, of course, but would be lovely to get, if possible. Below we describe the data we have been collecting, and that we will ask you to collect.

Methodology: To comply with ethics, data can be collected passively only at *public* events i.e. seminars that are advertised widely and can be attended by the public. Most departmental seminars fit into this category. Seminars within lab group probably do not count—please do not collect data at these.

We would like to ensure that our activities do not affect the behaviour of the audience (and affect the data we collect), ***so we please ask that you do not tell your colleagues about our study***.

The data we collect will be pooled into a google sheet "question_gender_groupsheet". Please click here to request access to a Google sheet for entering your data:

https://docs.google.com/spreadsheets/d/1pr2zj97e1hFHF-BqS49aHBX2csGS2r0infMlK49WdLY/edit?usp=sharing

The data to be collected during the talks are very straightforward, as is the google sheet. There is a greyed line of example data at the top of this sheet for guidance.

The column headings and data (* indicates data to be collected during the seminars, whereas all other data can be collected at another time) to be inputted are:

country: the country in which the talk took place

university: the university / institution in which the talk took place

department: the name of the department in which the talk took place. This is to control not only for differences between departments, but also so that we can retrospectively control for differences between subjects. We've been using short versions such as "Zoology" and "BioAnth" instead of "Department of Zoology" and "Department of Biological Anthropology".

observer: your name (will not be published! Just for queries)

speaker: the speaker's name (this information will not be shared, it is collected only so that we can collect further information on career stage, speakers' institutions etc. later if necessary)

speakerInternal: T or F. Is the speaker a member of your department? (To control for whether it is easier to ask questions of speakers with whom you are familiar.)

speakerSex: M or F for male or female, respectively

title: the title of the talk (again, so that we can retrospectively code the subject if necessary)

date: the date of the talk as DD/MM/YYYY

time: the time of the talk as HHMM e.g. 1630

***N_attendees**: number of attendees at the seminar. Includes the host (if any), and the observer (i.e. you), but not the speaker. Please be accurate—count twice (and more times if you have disagreement!). Many people arrive late i.e.

after the talk has started. We have been including these individuals as they are often obvious. However, we have NOT been reducing the totals for people who leave towards the end of the seminar.

***N_male**: the number of male attendees at the seminar. As for N_attendees, please count at least twice for either males or females or both.

***N_female**: the number of female attendees at the seminar.

What we define as a question: For the following data, there is a tricky <ahem> question of what constitutes an independent question. This is because the same attendee may ask two questions in a row, or an attendee may follow-up on/add to a question asked by another attendee, etc. As we are interested in visibility, a 'question' may be a comment or statement made by an attendee, rather than a question requiring an answer (though it usually does get a comment from the speaker). Follow-up questions (i.e. asking for *clarification* of the response of the speaker) are not included, but a subsequent question by the same attendee requiring a different response is included. We have thus defined a question as "*a statement made by an attendee that solicits a response from the speaker*". It is NOT the number of attendees who asked questions. It is thus feasible (but unlikely) that M/F_question could be greater than N_male / N_female. Questions that are asked during talks (i.e. the speaker is interrupted), are included, but are recorded in a separate column as we are interested in the gender of people who interrupt a talk (this is a rare occurrence for us, but may be more habitual elsewhere). Please include your own question should you ask one.

***First_question**: F or M. The gender of the attendee that asked the first question during the 'official' question period (i.e. after the talk has ended).

***M_question**: the number of questions asked by male attendees.

***F_question**: the number of questions asked by female attendees.

***M_during**: the number of questions asked by male attendees *during* the seminar (i.e. interrupting questions).

***F_during**: the number of questions asked by female attendees *during* the seminar (i.e. interrupting questions).

***question_time_start**: the time at which the questions start as HHMM e.g. 1650

***question_time_end**: the time at which the questions end as HHMM e.g. 1703

These two questions are to assess the duration of the question session and (roughly) for how long the speaker spoke.

The following questions are to control for the possible higher visibility of and (unconscious) preference given to more senior faculty in departments. We are defining *senior faculty* as the equivalents of University Teaching Officers, Academic Staff, etc. To collect these data in a standardised way, please go to the seminar department's website and collect these data from the "People" webpage (most departments have one) where they list "Academics" / "Teaching staff" / equivalent level of low-turnover, highly visible (e.g. teaching) staff.

M_faculty: the number of male senior faculty employed in the department

F_faculty: the number of female senior faculty employed in the department

We've made a small data collection sheet for you for the data that are to be collected during talks in case it helps (attached, with a particularly leading example in the top box). If you use any abbreviations, please be consistent so that these terms can be analysed straightforwardly in R.

Thanks very much for your help!

Alecia Carter
Dieter Lukas
Gillian Sandstrom
Alyssa Croft

Date:	16/06/2016	Speaker:	Dieter Lukas		
Talk title:	Mammals are awesome.				
N_attendees:	33	N_male:	17	N_female:	16
First_question:	M	M_question:	HHH /	F_question:	///
question_start_time	1720	question_end_time	1732	M_during:	/
				F_during:	–

Date:		Speaker:			
Talk title:					
N_attendees:		N_male:		N_female:	
First_question:		M_question:		F_question:	
question_start_time		question_end_time		M_during:	
				F_during:	

Date:		Speaker:			
Talk title:					
N_attendees:		N_male:		N_female:	
First_question:		M_question:		F_question:	
question_start_time		question_end_time		M_during:	
				F_during:	

Date:		Speaker:			
Talk title:					
N_attendees:		N_male:		N_female:	
First_question:		M_question:		F_question:	
question_start_time		question_end_time		M_during:	
				F_during:	
