# Supplementary material for: Women’s visibility in academic seminars: Women ask fewer questions than men
Source: PLoS One. 2018 Sep 27;13(9):e0202743. doi: 10.1371/journal.pone.0202743 (PMC6159863; doi:10.1371/journal.pone.0202743)
Supplement: S3 File — The tables show a more detailed breakdown of the survey data by gender than what is reported in the text. Included are tables that break down the answers to the questions about (1) why respondents sometimes do not ask questions when they have one; (2) if they believe that women ask fewer questions, why they think that women do not ask questions; and (3) factors they think may be important in encouraging women to ask questions after academic seminars. (PDF) [file pone.0202743.s003.pdf]

S3 File for: Women's visibility in academic seminars: women ask fewer questions than men

Alecia Carter, Alyssa Croft, Dieter Lukas, Gillian Sandstrom

Supplementary Table A:
Responses of a sample of academics who identify as male and female about what factors prevent them from asking a question after a seminar, even when they had a question to ask

		Reported importance					Kruskal-Wallis test	
**Reason** / **Gender**	**N**	**Not at all (N, prop)**	**Slightly (N, prop)**	**Moderately (N, prop)**	**Very (N, prop)**	**Extremely (N, prop)**	χ^2	***P***
Couldn't work up the nerve							16.63	<0.001
Female	277	39 (0.14)	56 (0.20)	52 (0.19)	77 (0.28)	53 (0.19)		
Male	188	54 (0.29)	36 (0.19)	40 (0.21)	38 (0.2)	20 (0.11)		
The speaker was too eminent/intimidating							17.15	<0.001
Female	275	83 (0.30)	92 (0.33)	60 (0.22)	31 (0.11)	9 (0.03)		
Male	188	88 (0.47)	59 (0.31)	29 (0.15)	9 (0.05)	3 (0.02)		
Not my field							4.78	0.03
Female	276	44 (0.16)	79 (0.29)	70 (0.25)	67 (0.24)	16 (0.06)		
Male	186	46 (0.25)	54 (0.29)	43 (0.23)	28 (0.15)	15 (0.08)		
Worried that I was not clever enough to ask a good question							21.34	<0.001
Female	276	55 (0.20)	58 (0.21)	62 (0.22)	52 (0.19)	49 (0.18)		
Male	188	75 (0.40)	36 (0.19)	33 (0.18)	27 (0.14)	17 (0.09)		
Worried that I had misunderstood the question							17.29	<0.001
Female	276	18 (0.07)	50 (0.18)	60 (0.22)	96 (0.35)	52 (0.19)		
Male	187	19 (0.10)	59 (0.32)	36 (0.19)	59 (0.32)	14 (0.07)		
Not sure whether the question was appropriate							12.87	<0.001
Female	276	21 (0.08)	52 (0.19)	89 (0.32)	80 (0.29)	34 (0.12)		
Male	187	27 (0.14)	49 (0.26)	54 (0.29)	49 (0.26)	8 (0.04)		

I was meeting the speaker later / asked after the talk had ended							2.02	0.16
Female	273	73 (0.27)	71 (0.26)	63 (0.23)	51 (0.19)	15 (0.05)		
Male	187	46 (0.25)	33 (0.18)	58 (0.31)	38 (0.20)	12 (0.06)		
Not enough time							3.96	0.05
Female	273	38 (0.14)	78 (0.29)	74 (0.27)	67 (0.25)	16 (0.06)		
Male	188	24 (0.13)	43 (0.23)	48 (0.26)	50 (0.27)	23 (0.12)		

Presented are the questions; the numbers of respondents of each gender who answered the question (N); the numbers and proportions of each gender who responded that the indicated factor was not at all, slightly, moderately, very, and extremely important (N, prop) for preventing them from asking questions; and the results of a Kruskal-Wallis test (in all cases, df = 1) indicating whether there was a difference between the genders' responses, including the test statistic (χ^2) and significance (*p*).

Supplementary Table B:
Responses of a sample of academics who identify as male and female, and who indicated that they believed that men asked more questions than women, about what they believed prevented women from asking a question if they had one.

		Reported importance					Kruskal-Wallis test	
**Reason** **Gender**	***N***	**Not at all (N, %)**	**Slightly (N, %)**	**Moderately (N, %)**	**Very (N, %)**	**Extremely (N, %)**	**χ^2**	***P***
Can't work up the nerve							12.39	<0.001
Female	178	8 (0.04)	22 (0.12)	55 (0.31)	72 (0.4)	21 (0.12)		
Male	85	8 (0.09)	17 (0.2)	35 (0.41)	20 (0.24)	5 (0.06)		
Feel intimidated by the speaker							1.98	0.16
Female	178	14 (0.08)	41 (0.23)	65 (0.37)	45 (0.25)	13 (0.07)		
Male	83	9 (0.11)	24 (0.29)	27 (0.33)	20 (0.24)	3 (0.04)		
Feel they are not an expert							32.69	<0.001
Female	178	7 (0.04)	14 (0.08)	44 (0.25)	85 (0.48)	28 (0.16)		
Male	85	17 (0.2)	14 (0.16)	28 (0.33)	23 (0.27)	3 (0.04)		
Believe that they are not clever enough to ask a good question							16.05	<0.001
Female	178	14 (0.08)	37 (0.21)	44 (0.25)	63 (0.35)	20 (0.11)		

Male	84	21 (0.25)	17 (0.2)	26 (0.31)	17 (0.2)	3 (0.04)		
Worried that they misunderstand the content							43.93	<0.001
Female	178	13 (0.07)	35 (0.20)	60 (0.34)	56 (0.31)	14 (0.08)		
Male	84	32 (0.38)	19 (0.23)	26 (0.31)	6 (0.07)	1 (0.01)		
Are unsure that their questions are appropriate							18.15	<0.001
Female	178	7 (0.04)	35 (0.2)	53 (0.3)	67 (0.38)	16 (0.09)		
Male	85	19 (0.22)	19 (0.22)	25 (0.29)	19 (0.22)	3 (0.04)		
Ask questions after the seminar is over							21.24	<0.001
Female	175	15 (0.09)	46 (0.26)	53 (0.3)	42 (0.24)	19 (0.11)		
Male	80	23 (0.29)	24 (0.3)	22 (0.28)	9 (0.11)	2 (0.03)		

Presented are the questions; the numbers of respondents of each gender who answered the question (N); the numbers and proportions of each gender who responded that the indicated factor was not at all, slightly, moderately, very, and extremely important (N, %) for preventing women from asking questions; and the results of a Kruskal-Wallis test (in all cases, df = 1) indicating whether there was a difference between the genders' responses, including the test statistic (χ^2) and significance (*p*).

Supplementary Table C:
Responses of a sample of academics who identify as male and female about what factors would encourage them to ask more questions after a seminar

			Reported importance					Kruskal-Wallis test	
**Reason** / **Gender**	***N***	***M***	**Not at all (N, %)**	**Slightly (N, %)**	**Moderately (N, %)**	**Very (N, %)**	**Extremely (N, %)**	χ^2	***P***
Confidence								29.97	<0.001
Female	277	3.81	18 (0.06)	18 (0.06)	56 (0.20)	93 (0.34)	92 (0.33)		
Male	188	3.12	32 (0.17)	29 (0.15)	47 (0.25)	44 (0.23)	36 (0.19)		
A chance to ask in person								5.46	0.02
Female	276	3.57	10 (0.04)	24 (0.09)	78 (0.28)	126 (0.46)	38 (0.14)		
Male	186	3.35	10 (0.05)	25 (0.13)	59 (0.32)	73 (0.39)	19 (0.1)		
Seniority								41.19	<0.001
Female	275	3.48	23 (0.08)	29 (0.11)	71 (0.26)	97 (0.35)	55 (0.20)		
Male	187	2.71	41 (0.22)	42 (0.22)	47 (0.25)	44 (0.24)	13 (0.07)		

A longer time to formulate question								3.45	0.06
Female	276	2.76	28 (0.1)	74 (0.27)	122 (0.44)	41 (0.15)	11 (0.04)		
Male	187	2.57	32 (0.17)	49 (0.26)	78 (0.42)	23 (0.12)	5 (0.03)		
Moderator doing a better job engaging whole audience								5.65	0.02
Female	271	2.71	43 (0.16)	73 (0.27)	91 (0.34)	48 (0.18)	16 (0.06)		
Male	185	2.45	51 (0.28)	44 (0.24)	54 (0.29)	28 (0.15)	8 (0.04)		
Nicer speakers								2.31	0.13
Female	273	2.48	52 (0.19)	88 (0.32)	90 (0.33)	37 (0.14)	6 (0.02)		
Male	188	2.35	47 (0.25)	63 (0.34)	50 (0.27)	22 (0.12)	6 (0.03)		
More welcoming host								5.83	0.02
Female	274	2.44	64 (0.23)	82 (0.3)	80 (0.29)	40 (0.15)	8 (0.03)		
Male	185	2.20	54 (0.29)	69 (0.37)	37 (0.2)	21 (0.11)	4 (0.02)		
Having a moderator to ask the questions								24.03	<0.001
Female	276	2.49	60 (0.22)	80 (0.29)	92 (0.33)	30 (0.11)	14 (0.05)		
Male	188	2.01	85 (0.45)	46 (0.24)	35 (0.19)	15 (0.08)	7 (0.04)		

Presented are the questions; the numbers of respondents of each gender who answered the question (N); the mean (*M*) of the responses of each gender (ordered from highest to lowest mean, averaged across gender); the numbers and proportions of each gender who responded that the indicated factor was not at all, slightly, moderately, very, and extremely important (N, %) for encouraging them to ask more questions; and the results of a Kruskal-Wallis test (in all cases, df = 1) indicating whether there was a difference between the genders' responses, including the test statistic (χ^2) and significance (*p*).
