# Supplementary material for: Women’s visibility in academic seminars: Women ask fewer questions than men
Source: PLoS One. 2018 Sep 27;13(9):e0202743. doi: 10.1371/journal.pone.0202743 (PMC6159863; doi:10.1371/journal.pone.0202743)

S4 File for: Women's visibility in academic seminars: women ask fewer questions than men

Alecia Carter, Alyssa Croft, Dieter Lukas, Gillian Sandstrom

More senior researchers (faculty) reported asking questions after seminars at higher frequencies than more junior researchers (post-graduate students and postdoctoral researchers; because of the small sample of undergraduate (*N* = 12), research fellow (*N* = 26), and "other" (*N* = 27) respondents, these individuals' responses have been excluded from these analyses) (linear model with frequency of asking questions as the response and career stage as a predictor (faculty as the reference category): post-graduate students β ± S.E. = -0.61 ± 0.10, *t* = -5.95, *p* <0.001; postdoctoral researchers β ± S.E. = -0.40 ± 0.12, *t* = -3.28, *p* = 0.001; Fig 1a). However, these data show that men self-report asking questions more frequently than women (LM with women as the reference category: β ± S.E. = 0.38 ± 0.09, *t* = 4.06, *p* <0.001; Fig 1b) and that this relationship holds at the same rate at every career stage (no significant interaction between gender and career stage; Fig 1c).

Supplementary Fig A: The relationship between seniority and gender on the self-reported frequencies of question asking behaviour of the survey respondents.

Shown are the proportions of respondents who report asking questions at seminars (a) at different career stages (pgrad = post-graduate student; pdoc = postdoctoral researcher; fac = faculty); (b) for respondents who identified as female and male; and (c) for female (f) and male (m) respondents at each career stage.

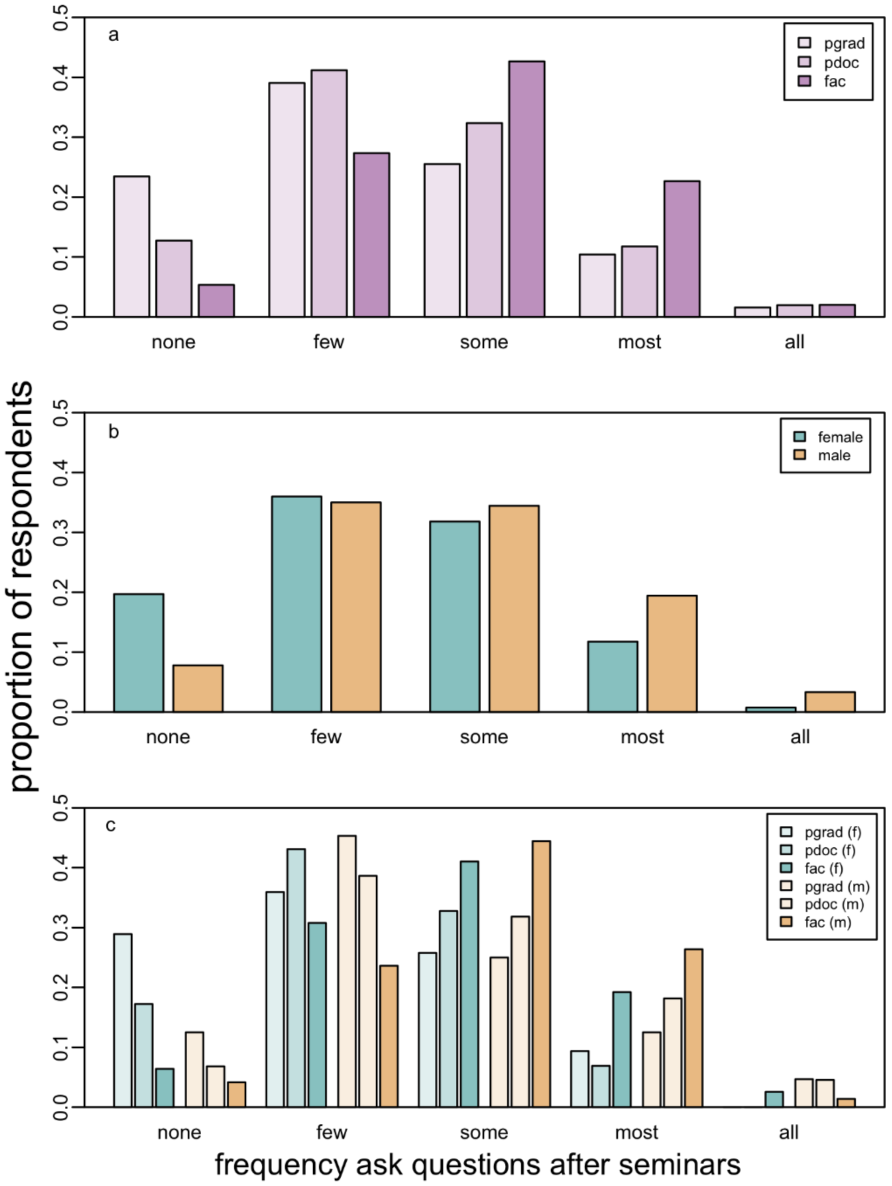

Supplement: S4 File — The results of linear mixed models are presented that test for relationships between survey respondents’ gender and seniority on their self-reported frequency of asking questions. A figure is also included that reflect these data. (PDF) [file pone.0202743.s004.pdf]
